# Negative Selection on *BRCA1* Susceptibility Alleles Sheds Light on the Population Genetics of Late-Onset Diseases and Aging Theory

**DOI:** 10.1371/journal.pone.0001206

**Published:** 2007-11-21

**Authors:** Samuel Pavard, C. Jessica E. Metcalf

**Affiliations:** 1 Laboratory of Survival and Longevity, Max Planck Institute for Demographic Research, Rostock, Germany; 2 Groupe d'Éco-Anthropologie, Museum National d'Histoire Naturelle, Paris, France; 3 Duke Population Research Institute, Duke University, Durham, North Carolina, United States of America; University of Utah, United States of America

## Abstract

The magnitude of negative selection on alleles involved in age-specific mortality decreases with age. This is the foundation of the evolutionary theory of senescence. Because of this decrease in negative selection with age, and because of the absence of reproduction after menopause, alleles involved in women's late-onset diseases are generally considered evolutionarily neutral. Recently, genetic and epidemiological data on alleles involved in late onset-diseases have become available. It is therefore timely to estimate selection on these alleles. Here, we estimate selection on *BRCA1* alleles leading to susceptibility to late-onset breast and ovarian cancer. For this, we integrate estimates of the risk of developing a cancer for *BRCA1*-carriers into population genetics frameworks, and calculate selection coefficients on *BRCA1* alleles for different demographic scenarios varying across the extent of human demography. We then explore the magnitude of negative selection on alleles leading to a diverse range of risk patterns, to capture a variety of late-onset diseases. We show that *BRCA1* alleles may have been under significant negative selection during human history. Although the mean age of onset of the disease is long after menopause, variance in age of onset means that there are always enough cases occurring before the end of reproductive life to compromise the selective value of women carrying a susceptibility allele in *BRCA1*. This seems to be the case for an extended range of risk of onset functions varying both in mean and variance. This finding may explain the distribution of *BRCA1* alleles' frequency, and also why alleles for many late-onset diseases, like certain familial forms of cancer, coronary artery diseases and Alzheimer dementia, are typically recent and rare. Finally, we discuss why the two most popular evolutionary theories of aging, mutation accumulation and antagonistic pleiotropy, may underestimate the effect of selection on survival at old ages.

## Introduction

Evolutionary theories of senescence (i.e. the increasing risk of death at older ages) are based on the fact that selection pressures on mortality diminish with age. This is because genes expressed at a specific age only affect individuals which survive to this age, who account for a decreasing proportion of the population as age increases. These theories assume therefore that mutations increasing mortality late in life are either subject: 1) to very small negative selection and can therefore accumulate (mutation accumulation) or 2) to small enough negative selection to be traded-off against higher fertility or survival at young ages (antagonistic pleiotropy). In both cases, deleterious mutations acting at older ages will persist and their cumulative effects on mortality will be responsible for senescence. Theoretical models of these alternatives calculate selection pressures on mortality as a change in fitness relative to a change in mortality at a specific age [1], or over a specific range of ages [2], [3], [4]. Allele frequency distributions that would result if selection and mutation were in balance for age-specific alleles have also been provided [3], [5]. Genetic research has identified few alleles involved in decreasing human mortality at old ages [6] but thousands of deleterious alleles are linked to susceptibility to late-onset diseases. For example, the Online Mendelian Inheritance in Man Database (OMIM, see www.ncbi.nlm.nih.gov) records dozens of late-onset diseases linked to genetic susceptibility; sometimes involving a single gene (e.g. Huntington's disease) but often numerous genes (e.g. Alzheimer's). Epidemiological data on the age of onset of such genetic diseases are now available. Surprisingly, no study has used these data to estimate selection against late-acting deleterious mutations. However, this would be informative because, although theoretical models assume a constant change in mortality at a given age or across a window of ages [1], [2], [4], genetic diseases show complex patterns of variability in age of onset. Additionally, assessing whether a mutation has been selected against in past human history requires that selection be estimated in realistic demographic scenarios: human demography varies considerably between populations which can affect both the strength of selection and genetic drift.

Genetic susceptibility to breast and ovarian cancer in women arising from a mutation in the *BRCA1* gene is one of the most widespread genetic diseases. Such mutations occur in 1 out of 3000 women in the USA [7], although frequencies vary considerably among populations [8]. Women with a mutation have a 65% risk of developing breast cancer before age 70 [9]. Breast and ovarian cancers linked to mutations in *BRCA1* are therefore likely to have been one of the main genetically-related causes of death in middle-aged women in past populations. *BRCA1* mutations can be therefore regarded as important deleterious mutations involved in old age mortality. It is consequently of interest to estimate selection on *BRCA1* mutations in relevant demographic scenarios for the light it can shed on senescence.

From the perspective of epidemiological genetics, *BRCA1* is remarkable being highly polymorphic with both rare and frequent alleles [8]. In 2006, the Breast Cancer Information Core (BIC, see www.nhgri.nih.gov/Intramural_ research/Lab_transfer/Bic) recorded more than 1000 alleles involved in a significantly higher susceptibility of carriers to develop a breast or ovarian cancer. Moreover, the frequency distribution of these alleles is highly heterogeneous [8]. Because the mean age of onset of breast and ovarian cancer is long after menopause [9], when selection is considered nonexistent, *BRCA1* alleles are generally thought to be selectively neutral. Mutation frequencies have therefore been explained by founder effects and population history [8]. Some mutations accord well with this explanation: for example the allele 185delAG is found in more than 1% of Ashkenazi Jewish women [10]. However, prevalence of breast cancer is not only associated with a few frequent mutations resulting from a founder effect, but also with many rare mutations. For example in Italy, most *BRCA1* mutations are at low frequencies and even in most cases recorded in only one family [8]. More generally, Wright et al. [11] highlighted that most alleles involved in genetic diseases including familial forms of cancer (such as breast cancer linked to *BRCA1*), coronary artery diseases and Alzheimer dementia, are recent and rare. These are characteristics of alleles involved in early onset diseases and subject to selection. Despite the fact that these diseases generally act after menopause, we would therefore expect these alleles to show direct or indirect effects on fitness.

In this paper we aim to determine whether *BRCA1* mutations have been neutral or under negative selection in recent human history. To do this, we incorporate estimates of age of onset of cancer [9] into a population genetic framework under different demographic scenarios. This is important because selection will depend on the proportion of women surviving at ages at which they are susceptible to develop the disease and the number of offspring produced by these women. To generalize our conclusions to diseases characterized by different patterns of age of onset we also explore the effect of changes in the mean and variance of age of onset on selection. This required that we 1) obtain parameters for the hazard of developing cancers for *BRCA1* mutation carriers; 2) obtain demographic parameters of fertility and survival for the populations considered; 3) determine the mortality hazard of mutation carriers and non-carriers in these populations; and 4) integrate these hazards into a population genetics framework to estimate the magnitude of selection on mutations in *BRCA1*. We outline these steps below (the code in ‘R’ allowing calculations for steps 1,3 and 4 is provided in the supplementary material entitled Code S1).

## Methods

### Hazard of Developing Cancers for BRCA1 Mutation Carriers

**Figure 1:**
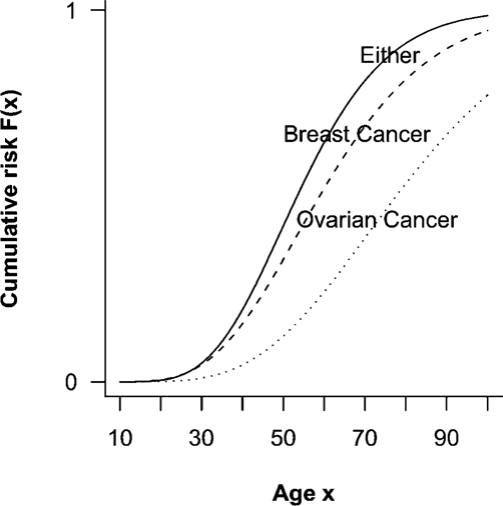
Age-specific risk of developing the disease. Women's cumulative risk of developing breast cancer (dashed line, *F_b_*(*x*)), ovarian cancer (dotted line, *F_o_*(*x*)) or either (solid line, *F_bo_*(*x*)) in a population free of any other causes of death. The two functions *F_b_*(*x*) and *F_o_*(*x*) are fitted 2-parameter cumulative gamma distributions from data from [9]. For breast cancer, *F_b_*(*x*), *α* = 7.93 and *β* = 7.54; and for ovarian cancer, *F_o_*(*x*), *α* = 8.20 and *β* = 9.86. The cumulative gamma function associated with this risk *F_bo_*(*x*) has parameters *α* = 9.97 and *β* = 5.54.

We described the distribution of the age of onset of cancer for women carrying a *BRCA1* mutation by a cumulative distribution function *F*(*x*), (i.e. the cumulative risk of developing a cancer before age *x*, *p*(*X*≤*x*)). We used data from a recent meta-analysis of 22 studies [9] to obtain the cumulative risk with age *x* of developing breast cancer, *F_b_*(*x*), and ovarian cancer *F_o_*(*x*), for individuals carrying a *BRCA1*-mutation in a population free of any other causes of death. This risk is an average over all *BRCA1* susceptibility alleles. As the risk of developing a breast or an ovarian cancer increases between age 20 and 49 and then decreases up to age 69 (no data are available after this age, [9]), we modeled the cumulative risks *F_b_*(*x*) and *F_o_*(*x*) by a 2-parameter cumulative gamma distribution 
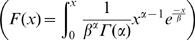
 fitted using a non-linear least squares approach from [9]. The corresponding hazards over ages *h_b_*(*x*) and *h_o_*(*x*) were estimated using the identity *h*(*x*) = *F*′(*x*)/(1−*F*(*x*)), where *F*′(*x*) is the probability density function obtained as the derivative of the cumulative risk function *F*(*x*). Under the assumption that both risks of developing a breast or an ovarian cancer are independent for mutation carriers, we estimated the hazard *h_bo_*(*x*) of developing either breast or ovarian cancer using the competing risks equation *h_bo_*(*x*) = *h_b_*(*x*)+*h_o_*(*x*). The corresponding cumulative risk *F_bo_*(*x*) is given by the identity and was also fitted by a 2-parameter gamma function (Figure 1). The mean and variance of age of onset associated with this curve are *μ* = *αβ* = 55 and *σ* ^2^ = *αβ* ^2^ = 306. As the hazard *h_bo_*(*x*) is considerably higher for individuals carrying a mutation than for individuals without a mutation (up to 61 times higher), the risk for carriers of developing a sporadic cancer (i.e. not associated with a mutation) can be considered negligible. We therefore defined *h_bo_*(*x*) as the hazard associated with the risk of developing a non-sporadic breast or ovarian cancer.

### Fertility and Survival for Three Demographic Scenarios

**Figure 2:**
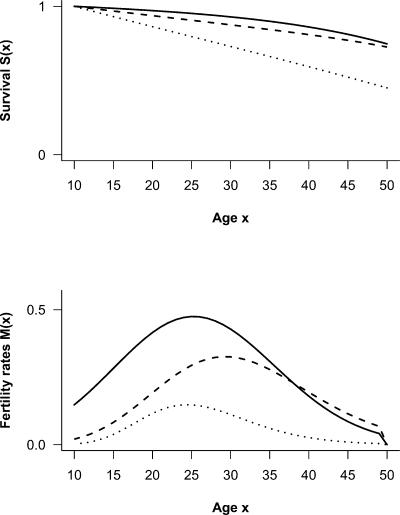
Demographic scenarios. Adult women's survival (A) and fertility (B) from ages 10 to 50 for the population of early Quebec (solid line), the Ache (dashed line) and the modeled population (dotted line). These populations differ by their growth (respectively high, intermediate and stationary, see [12], [13], [14]). Female adults' age-specific survivals were modeled by a Gompertz-Makeham. For Quebec, *a* = 4.69×10^−4^, *b* = 7.28×10^−2^, Δ = 0 and *c* = 1.49×10^−3^. For the Ache, *a* = 3.00×10^−5^, *b* = 1.10×10^−1^, Δ = 0 and *c* = 6.33×10^−3^. For the modeled population, *a* = 1.99×10^−3^, *b* = 5.82×10^−2^, Δ = 10 and *c* = 1.2×10^−2^. Female adults' fertilities were modeled by a Hadwiger function. For Quebec, *TFR* = 12.25, θ_1_ = −246.92, θ_2_ = 18.72, θ_3_ = 272.77. For the Ache, *TFR* = 8.032, θ_1_ = −36.82, θ_2_ = 4.79, θ_3_ = 68.29. For the modeled population *TFR* = 2.578, θ_1_ = −7.61, θ_2_ = 3.31, θ_3_ = 34.31.

If current allele frequencies are due to negative selection on *BRCA1*-mutations, this negative selection occurred in the past. We therefore used females' mortality and fertility estimates for different populations representing relevant demographic scenarios: 1) the population of early Quebec, 17th to 18th century French Canadians [12, pp. 131 and 88]; 2) the Ache, a population of hunter-gatherers from the Amazonian forests of Latin America [13, pp. 196 and 261] and 3) a theoretical stationary population with low life-expectancy and low fertility intended to capture human demography under extremely harsh conditions (Population type West, mortality level 1 and mean age at maternity in absence of death equals 27 years from [14, pp. 8 and 30]). These three demographic scenarios cover a wide range of demographic parameter space for adult survival and fertility. Their attributes can be summarized by two variables: remaining life-expectancy at age 10 of survivors at this age, 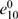; and mean number of daughters born to a woman surviving at age 50, Gross Replacement Rate (*GRR*). The population of Quebec has high adult survival and very high fertility 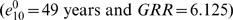; the Ache population has intermediate adult survival and fertility 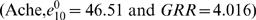 and the modeled population is an extreme case of a stationary population with very low adult survival and fertility 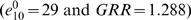. Data from life- and fertility-tables [12], [13], [14] were fitted using a non-linear least squares approach. As children with a mutation in *BRCA1* do not develop cancer before the onset of reproduction, and as fertility is zero after age 50, survival before 10 and after 50 will not alter the selection coefficient. Adult women's survival was therefore modeled from age 10 to age 50 using a Gompertz-Makeham function, 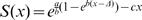, (Figure 2A).

We modeled fertility rates *M*(*x*) by a Hadwiger function [15], 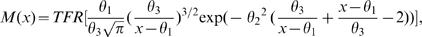
(1)where *TFR* is the total fertility rate estimated from data and where θ_1_, θ_2_, and θ_3_ have no particular demographic interpretation (but see [16] for calculation of the mean, the variance and the median of the density function for a Hadwiger distribution). This function was fitted for fertility rates between ages 10 and 49 and equal to 0 for younger and older ages respectively (Figure 2B). The Hadwiger function is better suited than the Brass function, for example, as it better captures late life fertility in pre-industrial populations [16].

### Mortality Hazard of Mutation Carriers and Non-Carriers

We needed to establish survival of *BRCA1* mutation carriers and non-carriers in the three demographic scenarios considered. In any population, the overall mortality hazard at age *x*, *h^pop^*(*x*), is the sum of the proportion of non-carriers *π^NC^*(*x*) times their mortality hazard *h^NC^*(*x*) and the analogous product for carriers *π^C^*(*x*)*h^C^*(*x*) defined as:
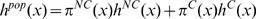
(2)where *π^NC^*(*x*) and *π^C^*(*x*) are the proportion of non-carriers and carriers in any age-class *x* such that *π^NC^*(*x*)+*π^C^*(*x*) = 1; and *h^NC^*(*x*) and *h^C^*(*x*) are their respective mortality hazards at age *x*. To establish the mortality hazard of mutation carriers for integration into the considered demographic contexts, we first assumed that all individuals who develop a cancer die in the absence of modern medicine. The hazard of developing a breast or ovarian cancer *h_bo_*(*x*) is therefore a mortality hazard. Note that the data used to obtain the corresponding cumulative risk of death of mutation carriers *F_bo_*(*x*) above is based on age at diagnosis which is necessarily earlier than the expected age at death. Our analysis does not distinguish these ages and therefore underestimates the mean age of death. Second, we assumed that there is no interaction between genes and the environment: i.e., the hazard of carriers *h_bo_*(*x*) estimated from [9] would be the same if estimated in any other environment. Third, we assumed independence between the risk of dying of a cancer resulting from a *BRCA1* mutation and the risks of any other causes of death (i.e. the corresponding mortality hazards are independent). With these three assumptions, *h_bo_*(*x*) can be added to the mortality hazard of any other causes of death in the population considered (denoted *h_a_*(*x*)) to calculate the overall mortality hazard of *BRCA1* mutation carriers, *h^C^*(*x*) = *h_bo_*(*x*)+*h_a_*(*x*). As non-carriers do not experience the mortality risk associated with non-sporadic breast or ovarian cancer hazard *h_bo_*(*x*), their overall mortality hazard *h^NC^*(*x*) is simply *h_a_*(*x*). Equation 2 becomes:
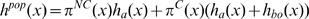
(3)The mortality hazard observed for the populations considered includes death of both carriers and non-carriers. In a heterogeneous population containing both non-carriers and carriers, the proportion of carriers *π^C^*(*x*) in a cohort will change with time due to their higher mortality so that there are increasingly fewer carriers at higher ages. This is a problem because, at any age, the mortality hazard observed in the population depends on the proportion of carriers and non-carriers at this age and these proportions are not known. However, except for populations with founder effects, the prevalence of cancer due to genetic susceptibility linked to *BRCA1* is unlikely to be greater than 1 in 3000 [8]. The proportion of carriers relative to non-carriers can therefore be for simplicity considered negligible at all ages (*π^NC^*(*x*)>>*π^C^*(*x*) for any *x*). The overall mortality hazard of the population *h^pop^*(*x*) provides us therefore with that of non-carriers *h^NC^*(*x*):
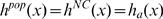
(4)Consequently, for any population considered, the mortality hazard of non-carriers is simply *h^NC^*(*x*) = *h^pop^*(*x*), and the mortality hazard of carriers is *h^C^*(*x*) = *h^pop^*(*x*)+*h_bo_*(*x*).

### Estimation of the Magnitude of Selection on Mutations in BRCA1

We needed to insert the demographic parameters obtained above into a population genetics framework to estimate selection. *BRCA1* is a tumor suppressor gene. A germline mutation in *BRCA1* occurs when one copy of the gene in a pair of homologues experiences a loss of function. A somatic mutation leading to the loss of function of the second gene in the pair completes the loss of function and renders the gene ineffective (i.e., the Loss of Heterozygocity, [17]). This second somatic mutation seems inevitable [17]: transmission of a single germinal copy of the gene is sufficient to result in predisposition to cancer. Genetic transmission of susceptibility to breast or ovarian cancer linked to the locus *BRCA1* is therefore dominant. We assumed that women carrying mutations in *BRCA1* only differ from those without by a higher mortality hazard *h^C^*(*x*)>*h^NC^*(*x*), i.e., mutation does not alter fertility of carriers relative to non-carriers, and likewise fertility does not alter susceptibility to cancer for carriers. These hazards correspond to two different survival functions *S^C^*(*x*) and *S^NC^*(*x*) such that 
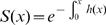
. The average number of children produced by a woman during her lifetime *W* can then be calculated as the sum over all ages of the product between the survival *S_x_* and fertility rates *Mx* for carriers and non-carriers respectively, 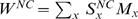 and 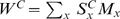. Assuming that the mutation is rare, the selection coefficient associated with the mutation is then given by 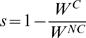. Negative selection against the mutation will largely override the effect of genetic drift if 2*Nes*>10, where *Ne* is effective population size (an estimate of the number of breeders), and *s* is the coefficient of selection defined above [18]. In other words, a deleterious mutation subject to negative selection such that 2*Nes*>10 will never go to fixation by chance through genetic drift, but will eventually be eliminated by selection. Having estimated *s*, we then calculated the smallest effective size required for the effect of negative selection to drive the mutation extinct (*Ne_min_* = 10/2*s*) and compared this with what it is known about the effective size of human populations to determine whether selection is strong enough to overcome the effect of genetic drift. *Ne* for Quebec is estimated at 1000 [19]. No estimates of *Ne* exist for the Ache or the modeled population, but human populations' *Ne* rarely falls below 100, with the exception of very isolated insular populations [20]. To explore implications of changes in the mean or variance of age of onset age for the selection coefficient, we altered both and calculated the resulting *s* and *Ne_min_*. This allowed us to test how robust our results are to our assumptions by exploring a range of parameter space; and to consider implications for a broader range of diseases with different mean and variance in age of onset.

## Results

**Figure 3:**
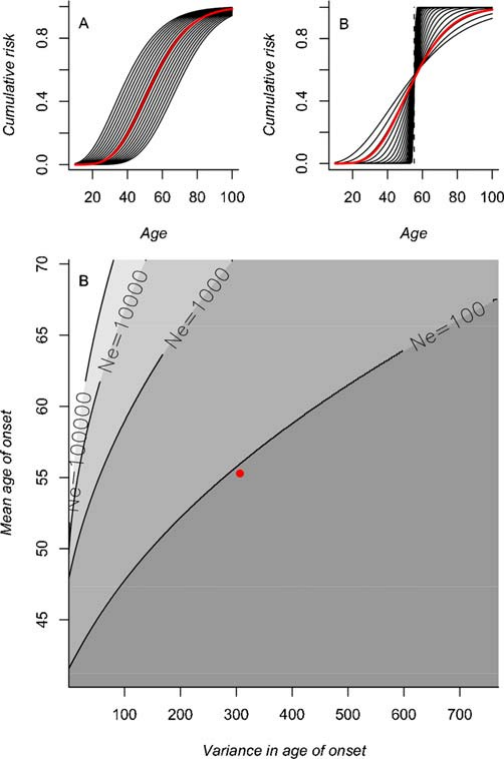
Selection on late-onset diseases. To explore a range of patterns of age of onset, we altered the observed cumulative risk with age *F_bo_*(*x*) of developing either breast or ovarian cancer for females carrying a *BRCA1* susceptibility allele estimated from [9] (red line) by changing the mean from age 40 to 70 (A) and the variance from 100 to 800 (B). This was achieved by altering parameters *α* and *β* of the cumulative gamma. We calculated the minimum effective population size *Ne_min_* = 10/2s necessary for negative selection to dominate over drift [18] corresponding to *BRCA1*-alleles associated with each of these curves. These *Ne_min_* are shown in (C) for the Quebec population. The point represents values estimated from [9] for the cumulative risk of developing breast and ovarian cancer given a mutation in *BRCA1* (mean = 55; variance = 306; *Ne_min_* = 92).

We found that the minimum effective population size *Ne_min_* such that selection against *BRCA1* mutations dominates over drift is 92 (*s* = 0.0541) for Quebec populations, 60 (*s* = 0.0833) for Ache populations and 130 (*s* = 0.0382) for the modeled population. Most human populations will generally have higher effective population sizes than these minimum effective sizes [20]. Consequently, despite the fact that *BRCA1* alleles are classically considered neutral due to their late age of onset, there is actually strong selection against them. This is because variance in age of onset means that younger individuals also develop the disease. Although most carriers develop the disease after age 45, 27% (Quebec), 24% (Ache) and 15% (modeled population) of breast and ovarian cancer cases occurred before this age. Figure 3 shows the minimum effective size *Ne_min_* required for selection to overcome genetic drift for mutations corresponding to a range of altered means (Figure 3A) and variances (Figure 3B) of the cumulative risk *F_bo_*(*x*) estimated for *BRCA1* alleles in the case of the population of Quebec (red line in Figure 3A and 3C). For most of the range explored, alleles experience strong negative selection (*Ne_min_*<1000) (Figure 3C). Results also show strong negative selection against high variance in the age of onset. Although the magnitude of selection fades quickly for very high mean and very low variance, the abundance of early cases is sufficient to lead to significant negative selection even for mean ages of onset occurring far beyond reproductive cessation (e.g. up to age 70 for the observed variance and for *Ne*<1000).

This also means that our results are robust to the assumptions of our analysis (independence between risk of breast cancer and ovarian cancer, certain death for individuals who develop a cancer, no interaction between the risk of non-sporadic cancer for carriers and the environment, independence between the risk of cancer and the risk of any other causes of death). Violation of the assumptions may change the mean and variance of the risk for carriers, but the magnitude of change is unlikely to extend beyond the range explored in our analysis (i.e. 15 years around the mean and a variance spanning from 100 to 800), which is predominantly in the area where selection overcomes drift (i.e. *Ne_min_*<1000).

**Figure 4:**
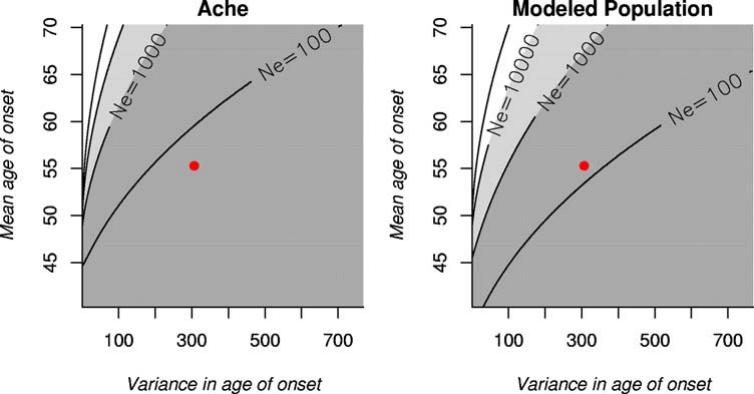
Selection on late-onset diseases for the Ache and the modeled population. As in Figure 3C but using survival and fertility for Ache (left panel) and the modeled population (right panel).

The Ache population and the modeled population show very similar selective patterns to those presented in Figure 3C (presented in Figure 4 for information). Results are therefore largely independent of the demographic scenario considered; even when mortality is very high and late fertility is low. This indicates that selection has occurred throughout human history. However, although the magnitude of selection on alleles is strong enough to overcome genetic drift in all three demographic scenarios considered, this magnitude nevertheless varies. This will affect the time to extinction of the mutation but not its fate. We discuss below (see discussion) which demographic traits most shape these differences in magnitude of negative selection. Finally these results do not include intergenerational transfers and negative selection is thereby underestimated: extending the model to incorporate the fact that women might contribute to their children's or grand-children's survival through post-natal maternal or grand-maternal care would strengthen our results [21].

## Discussion

### Population genetics of late-onset-diseases

We demonstrate that variance in age of onset strongly influences selection on alleles involved in susceptibility to late-onset diseases that lead to rapid death of carriers. For genetic diseases occurring in women and characterized by a mean age of onset long after menopause, cases generally do not occur at a specific age but are spread across several ages. Consequently, the proportion of cases arising during reproductive ages may not be negligible with respect to selection. Using a cumulative risk function averaged over all *BRCA1* alleles, we show that this pattern has resulted in negative selection on ’the average’ *BRCA1* alleles for three different demographic scenarios, from fast growing populations (high survival and fertility) to stationary populations (low survival and fertility): although the mean age of onset occurs after menopause (55 years old) between 15 and 27% of cases occur before age 45, allowing negative selection to operate. We therefore show 1) that negative selection on *BRCA1* alleles is likely to have operated during human history and 2) that negative selection is likely to apply for most alleles leading to late-onset diseases, as it occurs across a broad range of mean and variance of age of onset. For example, any other allele associated with a distribution of age of onset with a similar variance to that observed for *BRCA1* would be significantly negatively selected against even for a mean age of onset of up to 70 in a population of effective size 1000 (Figure 3C).

#### Are the selection coefficients calculated compatible with observed BRCA1 alleles frequencies?

This can be explored by calculating allele frequencies expected when selection and mutation are in balance. Homozygous individuals carrying susceptibility alleles on both chromosomes die before birth. The selection coefficient for this genotype *s_homo_* is therefore 1. For heterozygotes, the selection coefficient *s_hetero_* is as calculated above. It can also be seen as the product of the partial dominance coefficient of the susceptibility allele *h* (here the partial dominance results mainly from Loss Of Heterozygosity) and the selection coefficient for homozygote carriers *s_homo_*. Consequently, *s_hetero_* = *s_homo_h* = *h*. In a simple mutation-selection balance model for partially dominant alleles (in a closed, panmictic, stationary population with an even sex-ratio), the estimated frequency of susceptible alleles *q* is *q* = *μ*/*s_hetero_*, where *μ* is the rate at which susceptibility alleles arise in a new individual. If ∼1 woman in a 1000 carries a germline mutation in *BRCA1* at birth (*q* = 1×10^−3^), and if these mutations are negatively selected with a selection coefficient equal to that calculated for Quebec population (*s* = 0.0541), then the expected deleterious mutation rate *μ* = *qs* is 5×10^−5^ germline mutations per new individual. This mutation rate is in the range of those expected for most coding-protein genes [22].

#### How do different demographic scenarios alter estimates of selection?

Results remain consistent across a range of mortality and fertility schedules broad enough to incorporate most demographic scenarios possible through *Homo sapiens* history. Although the magnitude of selection varies from one to two fold between the modeled population and the Ache (*Ne_min_* equals 60 for the Ache and 130 for the modeled population), negative selection remains in a range likely to be efficient in most human populations [20]. This consistently strong selection arises because the proportion of children born to women at the end of their reproductive life relative to their lifetime reproductive success is not negligible in all three demographic scenarios considered (the proportion of children born to women older than age 39 is 6% of the adults' lifetime reproductive success for Quebec, 11% for the Ache and 2% for the modeled population). To assess the importance of fertility rates on selection on *BRCA1* susceptibility alleles, we calculated the *Ne_min_* for populations with the survival trajectory of Quebec and the Ache respectively, but the fertility of the modeled population (i.e. simulating a large decrease in fertility). *Ne_min_* rose to 112 for Quebec (from 92) and 113 for Ache (from 60). These differences are due to the fact that the population of Quebec and the Ache differ mainly through the higher late fertility rates observed in the Ache: the loss of late-fertility is therefore larger for Ache than for the population of Quebec. To assess the importance of survival, we also calculated the *Ne_min_* for populations with the fertility trajectory of Quebec and the Ache respectively, but survival of the modeled population (i.e. simulating a large decrease in survival). *Ne_min_* rose to 111 for Quebec (from 92) and 70 for Ache (from 60). These results indicate that changes in late fertility rates more strongly affect the magnitude of selection than changes in survival. Because large late fertility rates are likely to be a key demographic feature of past populations, we expect strong selection against alleles predisposing to late-onset diseases, even if survival at old ages was low.

Overall, these changes in populations' demography have a much lower impact on the fate of a mutation than the effective size of the population *Ne* (especially for alleles leading to high mean/low variance in age of onset, see Figure 3C and 4). Consequently, variation in the age-trajectories of survival and fertility among populations do not greatly alter selection on alleles involved in susceptibility to late-onset diseases (in agreement with results from [3]), unless such changes alter the effective population size. The latter is therefore likely to be the key variable affecting the purging of alleles involved in late onset diseases from human populations.

#### Can variation in the magnitude of selection explain the distribution of alleles frequencies observed for BRCA1?

Our results have implications for the genetic epidemiology of *BRCA1*. As *BRCA1* mutations have been selected against in past populations, negative selection may explain, with genetic drift and population history, the current geographic distribution of most *BRCA1* allele frequencies characterized by a large number of recent and rare alleles. However, why then are certain *BRCA1* alleles, some of which are known to have appeared more than 500 years ago still present [8], [23]? Founder effects have been identified in some cases (e.g. for the 189delAG mutation found in 1% of Ahskenazi Jews, which appeared 460–1600 years ago), but remain undetected for others (e.g. for 5382insC, the most common mutation in Russia and Europe, estimated age of appearance 360–1380 years ago, [8], [23]). A first explanation might be that these *BRCA1* mutations have positive pleiotropic effects on either fertility or early survival. However, the *BRCA1* protein is known to act in DNA repair [24], and other pleiotropic effects beyond preventing cancer onset are to date unknown. Alternatively, it is known that different mutations lead to different risks of developing cancer [25]. Old alleles, still present at relatively high frequencies, might be characterized by a higher mean age of onset and/or a lower variance and consequently be less negatively selected, facilitating their persistence through drift and migration. Another possibility is that the risk profile associated with alleles may have changed over the course of human history. Epidemiological studies have shown that women's reproductive trajectories (age at menarche and menopause, nulliparity, etc.) and nutrition are associated with variation in breast cancer risk. The interaction between BRCA1 susceptibility alleles and the environment or cultural behavior is still poorly understood [26] and empirically difficult to access [27], but inconsistencies between breast and ovarian cancer rates and mutation frequencies (e.g. [28]) provide some support for Eaton et al. 's notion that risks of cancer due to genetic susceptibility were lower in pre-industrial populations than in current populations [29]. Some susceptibility alleles may have therefore been under lower levels of negative selection in particular past environments, allowing them to reach higher frequencies. Our results are based on an estimation of the cumulative risk of developing cancers, averaged over all *BRCA1* mutations, and we therefore cannot differentiate the magnitude of selection acting on different alleles. Further study could assess if, among these alleles, alleles' frequencies are correlated with a higher mean and/or a lower variance in age of onset. If this prediction proved true, the frequency distribution of alleles could be used (with knowledge of population demographic history) to make predictions about the associated variation in risk of onset (between alleles and/or between environments); and vice versa.

### Aging theory

#### What do our results contribute to theories of aging?

Theoretical models consider that selection pressures on survival at old ages is small (and even null after menopause for women) because survival at these ages no longer affects fitness [1], [2], [3], [4]. We explored a range of parameter space corresponding to a large range of mean and variance in age of onset that might correspond to a variety of late onset diseases. Throughout this range, alleles were subject to considerable negative selection. This is firstly because age of onset of genetic diseases may be very variable, so that young individuals may die of the disease even for diseases with a late average age of onset. It is likely that very few genetic diseases affecting women late in life fail to affect at least some pre-menopausal individuals leading to selection where one might otherwise anticipate none. Secondly, a selection pressure does not indicate whether an allele is significantly selected against within a particular population. For a high enough effective population size, even small negative selection can be sufficient to eliminate an allele. For both reasons, the effect of selection on alleles involved in late onset diseases is likely to be generally under-estimated. Alleles considered neutral or nearly neutral (for mutation accumulation), or under negative selection small enough to be traded-off for a positive effect at younger ages (for antagonistic pleiotropy) may be more strongly negatively selected than thought. For antagonistic pleiotropy (for which there is the most empirical support, e.g., [30]), this implies that positive selection on alleles associated with increases of survival or fertility at young ages must be large enough to compensate for non-negligible negative selection on survival at old ages, highlighting the role of trade-offs in the evolution of senescence. For mutation accumulation, purging of the genetic burden may be more effective than hitherto suspected, suggesting a diminished role of mutation accumulation in determining mortality late in life.

Although our results suggest diminished importance of mutation accumulation in determining late-age mortality, they also provide a strong counter-argument to one of its major criticisms. Detractors of the theory of mutation accumulation argue that it is unrealistic as an explanation of senescence because hazard rates should go to infinity when the force of selection reaches zero (i.e. after menopause), resulting in a wall of death (i.e. an age beyond which there is no survival). Models incorporating intergenerational transfers [31] or models considering the genetic inter-dependence of males and females despite their demographic differences [32] produce a more gradual decrease in the force of selection after menopause, eliminating the possibility of this “wall of death” outcome. Additionally, here we show that simply considering genetically associated variance in the age of onset of late-onset diseases has a similar effect. In this respect, our results therefore provide support for the theory of mutation accumulation. Moreover, if our results prove true for a large number of diseases affecting women at old ages, variance in the age of onset for these diseases may explain extended post-reproductive life from a genetic, rather then behavioral or cultural basis.

#### Future directions

Initially, *BRCA1* appears a good candidate gene for testing the theory of mutation accumulation because 1) when all *BRCA1* mutations are taken together, the prevalence of breast and ovarian cancer linked to the *BRCA1* locus is one of the highest among late-onset diseases and 2) in the absence of modern medicine, the disease leads to rapid and certain death (as opposed to slowly degenerative diseases, like Alzheimer dementia, whose effects on fitness are unclear). However, we show that *BRCA1* mutations are subject to strong negative selection. *BRCA1* is therefore not a good candidate for mutation accumulation. This is also likely to be the case for other genetic susceptibilities where allele frequencies are characterized by a large number of recent and rare alleles, e.g. certain familial forms of coronary artery diseases and Alzheimer dementia. Further tests of the hypothesis of mutation accumulation might more fruitfully focus on diseases 1) epidemiologically characterized by a very late and/or less variable age of onset and/or 2) with an abundance of common and ancient alleles. We hope that our study will encourage further research using epidemiological data in a population genetics framework to test evolutionary theories of aging.

## Supporting Information

Code S1 This code in ‘R’ allows calculations for steps 1,3 and 4.
(0.05 MB DOC)
