# Supplementary material for: Negative Selection on BRCA1 Susceptibility Alleles Sheds Light on the Population Genetics of Late-Onset Diseases and Aging Theory
Source: PLoS One. 2007 Nov 21;2(11):e1206. doi: 10.1371/journal.pone.0001206 (PMC2065908; doi:10.1371/journal.pone.0001206)
Supplement: Code S1 — This code in ‘R’ allows calculations for steps 1,3 and 4. (0.05 MB DOC) [file pone.0001206.s001.doc]

# Program in 'R' for "Estimating Selection Against Late-Onset Diseases: the Case of BRCA1 in humans".

#############################################################################################################################

# The program has 4 sections.

# 1) Calculation of parameters for survival and fertility for the 3 populations considered: Quebec, Ache and Modelled population

# 2) Calculation of the hazard of BRCA1 alleles carriers (from data published by Antoniou and al., 2003).

# Given the population considered:

# 3) Calculation of the Net Reproductive Success (NRR) of Carriers, Non-Carriers, Selection Coefficient, Effective Size Ne and % of cancer cases arising before age 45

# 4) Alteration of the Mean and Variance of the Cumulative Risk of devlepping either a breast or an ovarian cancer for carriers and calculation of the corresponding minimum effective size for selection to overcome the effects of drift.

#Note that following the command "text(locator(1),"label-name") clicking on the open plot at the location where one desires the label-name to appear will cause this to happen

#############################################################################################################################

# 1) Survival and fertility for the 3 populations considered.

# 1.1) Survival (St) and hazard (haz): This is a model of survival from age 10 to infinity

#(although practically we are only interested in survival until age 50) of survivors at age 10.

# Fitted with a Gompartz-Makeham function (The parameter delta is used because

#the function for the 'Modelled population' converges better when the function is rescaled from age 0. such

# use of a delta parameter is a well-known method in demography for fitting Gompertz-Makeham functions

St<-function(x,a,b,Cste,Delta){

u<-exp((a/b)*(1-exp(b*(x-Delta)))-Cste*(x-Delta)) # Gompertz-Mahekam function for survival: St(10)~1

u[x<10]<-1 # St(x) is set to 1 for x<10 (individuals are surviving up to age 10)

return(u)}

haz<-function(x,a,b,Cste,Delta){

u<-a*exp(b*(x-Delta))+Cste # Gompertz-Mahekam function for hazard

u[x<10]<-0 # haz(x) is set to 0 for x<10 (individuals are surviving up to age 10)

return(u)}

# 1.2) Fecundity - 3 parameters Hadwiger function (1940)

fecundity<-function(x,varTFR,HAD1,HAD2,HAD3){

u<-varTFR*((HAD2/(HAD3*sqrt(pi)))*((HAD3/(x-HAD1))^(3/2))*exp((-HAD2^2)*((HAD3/(x-HAD1))+((x-HAD1)/HAD3)-2)))

u[x<10]<-0 # The function is fitted for fecundity =0 before age 10

u[x>49]<-0 # The function is fitted for fecundity =0 after age 49

return(u)

}

# 1.3) Parameters, choose the appropriate country for rest.

#Quebec

a=0.0004689606

b=0.0727611500

Cste=0.0014887440

Delta=0

TFR=12.25

HAD1= -246.91912

HAD2= 18.71655

HAD3= 272.77546

#Ache

a=2.998547e-05

b=1.104238e-01

Cste=6.326054e-03

Delta=0

TFR= 8.032

HAD1= -36.8166

HAD2= 4.795783

HAD3= 68.295291

# Modelled Population

a=0.001989644

b=0.058242830

Cste=0.012002329

Delta=1

TFR= 2.57708

HAD1= -7.611097

HAD2= 3.314885

HAD3= 34.311158

# 1.4) Remaining life-expectancy at age 10 of survivors at age 10 (10e10) and Gross Reproductive Rate (average number of daughters of females surviving to post-reproductive life, GRR)

e10=sum(St(10:100,a,b,Cste,Delta))

e10

GRR=sum(fecundity(1:100,TFR,HAD1,HAD2,HAD3))/2 # Note that these results slightly differ from those provided in the manuscript (in the part "Fertility and Survival for three demographic scenarios"). This is because, here are results provided by the fitted functions when we gave those from data in the manuscript.

GRR

# 1.5) Proportion of children born to women between age 40 and 50

sum(St(40:100,a,b,Cste,Delta)*fecundity(40:100,TFR,HAD1,HAD2,HAD3))/sum(St(10:100,a,b,Cste,Delta)*fecundity(10:100,TFR,HAD1,HAD2,HAD3))

#1.5) For Figure 2.

# Note that, for survival, the proportion of survivors at age 10 is here set to extactly 1 (because the resulting graph is more readable).

# However, this value could be any value between 0 and 1: Juvenile survival does not impact on the resulting selection coefficient since it is the same for both carriers and non carriers

par(mfrow=c(2,1),bty="l",las=1,mai=c(0.8,1.5,0.8,1.5),lab=c(10,1,7),pty="m", font.lab=2)

age<-seq(10,50,1)

plot(age,St(age,0.0004689606,0.0727611500,0.0014887440,0)/St(10,0.0004689606,0.0727611500,0.0014887440,0),type="l",lty=1,xlab="Age x", ylab="Survival S(x)",lwd=2,ylim=c(0,1), xlim=c(10,50))

points(age,St(age,2.998547e-05,1.104238e-01,6.326054e-03,0)/St(10,2.998547e-05,1.104238e-01,6.326054e-03,0),type="l",lty=2,lwd=2)

points(age,St(age,0.001989644,0.058242830,0.012002329,10)/St(10,0.001989644,0.058242830,0.012002329,10),type="l",lwd=2, lty=3)

age<-seq(10,50,1)

plot(age,fecundity(age,12.25,-246.91912,18.71655,272.77546), type="l",lty=1,xlab="Age x", ylab="Fertility rates M(x)",lwd=2,ylim=c(0,0.55))

points(age,fecundity(age,8.032, -36.8166, 4.795783, 68.295291),type="l",lty=2,lwd=2)

points(age,fecundity(age,2.57708, -7.611097, 3.314885, 34.311158),type="l",lty=3,lwd=2)

#############################################################################################################################

# 2) Calculation of the hazard of BRCA1 alleles carriers

# 2.1) Load the Data obtained in Antoniou et al.: Cumulative risk of developing a breast or ovarian cancer of carriers (obtained by digitizing the original information)

age<-c(20,30,40,50,60,70)

cum.risk.breast<-c(0, 0.2240896, 12.6610644, 38.2072829, 53.3333333, 65.0980392)/100 # Data for Breast cancer

cum.risk.ovar<-c(0.2240896, 0.5602241, 3.2492997, 14.3417367, 23.0812325, 38.8795518)/100 # Data for ovarian cancer

par(mfrow=c(1,1),pty="s",bty="l")

plot(age,cum.risk.breast,type="b",xlab="Age",pch=19, ylab="Cum risk")

points(age,cum.risk.ovar,col="red",type="b",pch=19)

# 2.2) Cumulative risk of developing a breast or ovarian cancer of carriers, modelled with a cumulative gamma distribution

agex<-seq(0,100,length=100)

##Gamma distribution

cum.gamma.func<-function(x,alpha,beta){

pgamma(x,alpha,beta)

}

# Model1: Cumulative risk of breast cancer

model1<-nls(cum.risk.breast ~ cum.gamma.func(age,alpha,beta),start=list(alpha=0.5,beta=0.001)) # Model1: Cumulative risk of breast cancer

coef(model1)

alph.est1<-coef(model1)[1]

beta.est1<-1/coef(model1)[2] # the value beta given in the paper (see fig.1) beta.est1 equals 1/(coeff. beta estimated by 'R')

alph.est1*beta.est1 # mean age of onset for Breast cancer

alph.est1*beta.est1^2 # variance of age of onset for Breast cancer

# Model2: Cumulative risk of ovarian cancer

model2<-nls(cum.risk.ovar ~ cum.gamma.func(age,alpha,beta),start=list(alpha=0.5,beta=0.001)) # Model2: Cumulative risk of ovarian cancer

coef(model2)

alph.est2<-coef(model2)[1]

beta.est2<-1/coef(model2)[2] # the value beta given in the paper (see fig.1) beta.est1 equals 1/(coeff. beta estimated by 'R')

alph.est2*beta.est2 # mean age of onset for Ovarian cancer

alph.est2*beta.est2^2 # variance of age of onset for Ovarian cancer

# 2.3) Cumulative risk of developing either a breast or an ovarian cancer for carriers of the mutation

hazard.cancer.carrier<-function(age,alpha1,beta1,alpha2,beta2){

fb<-dgamma(age, alpha1, rate = 1/beta1, log = FALSE)

fo<-dgamma(age, alpha2, rate = 1/beta2, log = FALSE)

Fb<-pgamma(age, alpha1, rate = 1/beta1, log = FALSE)

Fo<-pgamma(age, alpha2, rate = 1/beta2, log = FALSE)

hct<-(fb/(1-Fb))+(fo/(1-Fo)) # The hazard of developping either a breast or an ovarian cancer is the sum of the hazard (defined as f(x)/(1-F(x)))

return(hct)

}

# The cumulative risk of developing either a breast or an ovarian cancer at age x is therefore 1-exp( integral of the hazard of cancer for carriers from age 0 to age x )

# As the hazard is the sum between the ratio of a gamma distribution to 1- a cumulative gamma distribution, the integration is not trivial.

# We therefore solve it numerically:

NumSolve<-20000 # Decimal vector 'age.seq' coresponds to the number of steps where the hazard is estimated.

age.seq<-seq(1,100,length=NumSolve) # Definition of ages at which hazard is calculated

hazC<-(hazard.cancer.carrier(age.seq,alph.est1,beta.est1,alph.est2,beta.est2)) # Calculation of the hazard for these ages

hazC2<-hazC*100/NumSolve # Step-wise calculation of the hazard for each age

summedval<-rep(NA,NumSolve); summedval[1]<-hazC2[1] # Calculation of the cumulative hazard (called "summedval") for each of these age.

for (i in 2:NumSolve){

summedval[i]<-sum(hazC2[1:i])

}

surC<-exp(-summedval)

FOB<-1-surC # Survival= exp(- cumulative hazard)

# And fit the resulting distribution by a new gamma distribution: Cumulative risk distribution of developing either a breast or an ovarian cancer.

FOBmodelcum<-nls(FOB~ cum.gamma.func(age.seq,alpha,beta1),start=list(alpha=0.5,beta1=0.001))

coef(FOBmodelcum)

alpha.val<-coef(FOBmodelcum)[1]

beta.val<-1/coef(FOBmodelcum)[2]

mean.onset<-alpha.val*beta.val

var.onset<-alpha.val*beta.val^2

mean.onset # Mean age of onset (either a breast or ovarian cancer)

var.onset # Variance in age of onset (either a breast or ovarian cancer)

# 2.4) For Figure 1.

age<-10:100

par(mfrow=c(1,1),bty="l",font.lab=2)

plot(age,cum.gamma.func(age,alpha.val,1/beta.val), type="l",lty=1,xlab="Age x", ylab="Cumulative risk F(x)")

text(locator(1),"Either")

points(age,cum.gamma.func(age,alph.est1,1/beta.est1), type="l",lty=2)

text(locator(1),"Breast Cancer")

points(age,cum.gamma.func(age,alph.est2,1/beta.est2), type="l",lty=3)

text(locator(1),"Ovarian Cancer")

# 2.5) To compare the data and the model: Black (Breast), Red (Ovarian), Blue (Breast+Ovarian); point=observed values, lines=models, dot=numerical estimation

age<-c(20,30,40,50,60,70)

age2<-(NumSolve*age)/100

plot(age,cum.risk.breast,type="p",xlab="Age", ylab="Cum risk",pch=19) # Data Risk Breast

points(age,cum.gamma.func(age,alph.est1,1/beta.est1), type="l") # Model breast

points(age,cum.risk.ovar,type="p", col="red",pch=19) # Data Risk Ovarian

points(age,cum.gamma.func(age,alph.est2,1/beta.est2), type="l",col="red") # Model Ovarian

age2<-(NumSolve*age)/100

FOB2<-FOB[age2]

points (age,FOB2, col="blue", type="l",lty=3) # Numeric estimation of risk Breast+Ovarian

points(age,cum.gamma.func(age,alpha.val,1/beta.val), type="l",col="blue") # Model risk Breast+Ovarian

#############################################################################################################################

# 3) Calculation of the Net Reproductive Success of carriers and non-carriers; selection.coefficient and minimum effective size

#3.1) Non Carriers.

# Survival of carriers in the population is exp(-integral[hbo(t)+h(t)])=exp(-integral[hbo(t)]-integral[h(t)])=exp(-integral[hbo(t)])*exp(-integral[h(t)])=(1-Fob(t))*S(t)

age<-1:100

NRR.C<-sum(St(age,a,b,Cste,Delta)*(1-cum.gamma.func(age,coef(FOBmodelcum)[1],coef(FOBmodelcum)[2]))*fecundity(age,TFR,HAD1,HAD2,HAD3))

NRR.C

#3.1) Non Carriers.

age<-1:100

NRR.NC<-sum(St(age,a,b,Cste,Delta)*fecundity(age,TFR,HAD1,HAD2,HAD3))

NRR.NC

#3.2) Selection Coefficient and Effective Size

coeff.s<-1-NRR.C/NRR.NC

coeff.s

NeMin<-10/(2*coeff.s)

NeMin

#3.3) % of cancer occuring before age 45

perc<-sum(dgamma(1:45,coef(FOBmodelcum)[1],coef(FOBmodelcum)[2])*St(1:45,a,b,Cste,Delta))

perc

#3.4) For non-carriers, proportion of children born between age 40 and 50

sum(St(40:50,a,b,Cste,Delta)*(1-cum.gamma.func(40:50,coef(FOBmodelcum)[1],coef(FOBmodelcum)[2]))*fecundity(40:50,TFR,HAD1,HAD2,HAD3))/NRR.C

sum(St(40:49,a,b,Cste,Delta)*fecundity(40:49,TFR,HAD1,HAD2,HAD3))/NRR.NC

#############################################################################################################################

# 4) Change in Mean and Variance of the Cumulative Risk and calculation of the minimum effective size.

#4.1) Picture 3.A: Change in mean (ntest changes in the mean across a range from 55-15 to 55+15)

age<-10:100

ntest<-20

nf <- layout(matrix(c(1,2,3,3),2,2,byrow=TRUE), c(1,1,2), c(1,2), TRUE)

layout.show(nf)

deltMean<-seq(-15,15,length=ntest)

alph.new<-beta.new<-rep(NA,ntest)

for (i in 1:ntest){ # For each changes from change delMean[1] to change delMean[ntest]

alph.new[i]<-((sqrt(alpha.val)*sqrt(alpha.val*(beta.val^2))+deltMean[i])/(sqrt(alpha.val*(beta.val^2))))^2 # New Paramaters alpha of the gamma

beta.new[i]<-(mean.onset+deltMean[i])/alph.new[i] # New Paramaters beta of the gamma

}

#Plot out generated range of means

par(mar=c(4,4,1,1),pty="s",bty="l")

i<-1; plot(age,cum.gamma.func(age,alph.new[i],1/beta.new[i]),type="l",col="red",xlab=expression(italic("Age")), ylab=expression(italic("Cumulative risk")))

for (i in 1:ntest){

points(age,cum.gamma.func(age,alph.new[i],1/beta.new[i]),type="l")

}

#observed curve #

points(age,cum.gamma.func(age,alpha.val,1/beta.val),type="l",lwd=2,col="red")

legend(-15,1,legend="A",bty="n",lty=3)

#4.2) Picture 3.B: Change in variance (ntest changes in the variance ranging from 0.306 to 766)

deltVar<-exp(seq(log(0.001),log(2.5),length=ntest))

alph.new<-beta.new<-rep(NA,ntest)

for (i in 1:ntest){ # For each changes from change delVar[1] to change delVar[ntest]

var.wanted<-var.onset*deltVar[i] # Calculation of the new variance

beta.new[i]<-var.wanted/mean.onset # New Paramaters beta of the gamma

alph.new[i]<-var.wanted/(beta.new[i]^2) # New Paramaters alpha of the gamma

}

#Plot out generated range of variances

par(mar=c(4,4,1,1),pty="s",bty="l")

i<-1; plot(age,cum.gamma.func(age,alph.new[i],1/beta.new[i]),type="l",col="red",xlab=expression(italic("Age")), , ylab=expression(italic("Cumulative risk")))

points(c(alph.new[i]*beta.new[i],alph.new[i]*beta.new[i]),c(0,1),lty=2,type="l"); print(alph.new[i]*beta.new[i])

for (i in 1:ntest){

points(age,cum.gamma.func(age,alph.new[i],1/beta.new[i]),type="l")

}

#Add the observed curve (estimates from Antoniou et al., 2003) in red #

points(age,cum.gamma.func(age,alpha.val,1/beta.val),type="l",lwd=2,col="red")

legend(-15,1,legend="B",bty="n",lty=3)

#4.3) Calculation of selection coefficient coresponding to changes in mean and variance

ntest<-1000 # ntest is the number of change in mean and variance wanted

deltMean<-seq(-15,15,length=ntest) # Deviation to the mean for each change

deltVar<-exp(seq(log(0.001),log(2.5),length=ntest)) # Deviation to the variance for each change

mean.wanted<-mean.onset+deltMean # the resulting new mean for each change

var.wanted<-var.onset*deltVar # the resulting new variance for each change

age<-10:50 # Here again we are interested by change in mortality hazard during the reproductive life only, so from age 10 to age 50

store.coeff.s<-alph.new<-beta.new<-matrix(NA,ntest,ntest) # Set of the array of result

penet<-array(dim=c(ntest,ntest))#,1000))

for (i in 1:ntest){ # for any change in variance

for (j in 1:ntest){ # and for any change in mean

beta.new[i,j]<-var.wanted[i]/mean.wanted[j] # new parameter beta coresponding to the new gamma

alph.new[i,j]<-var.wanted[i]/(beta.new[i,j]^2) # new parameter alpha coresponding to the new gamma

NRR.C<-sum(St(age,a,b,Cste,Delta)*(1-pgamma(age, alph.new[i,j], rate = 1/beta.new[i,j], log = FALSE))*fecundity(age,TFR,HAD1,HAD2,HAD3)) # Calculation of the NRR of carriers

store.coeff.s[i,j]<-1-(NRR.C/NRR.NC) # Store the resulting selection coefficient

print(NRR.C)

}

print(i)

}

range(store.coeff.s)

#4.4) For Visualisation of these NeMin: Picture 3.C

store.coeff.s[store.coeff.s<0]<-0

store.coeff.s[is.na(store.coeff.s)]<-0

# preparing colors

brek<-c(-0.2,1/20000,1/2000,1/200,1/20,1/2, max(store.coeff.s,na.rm=T)+0.2)

colo <- gray((20:12)/20)

valsi<-c(8,7,6,5,4,3,2,1.5,1)

Nepic<-colo1 <- matrix(nrow=nrow(store.coeff.s), ncol=ncol(store.coeff.s)) # setting a matrix for the colors

# selecting the right color for the right position

# playing around with the positions in the data matrix

for(i in 1:length(colo)){

colo1[which(store.coeff.s>brek[i] &

store.coeff.s<=brek[i+1],

arr.ind=T)] <- colo[i]

Nepic[which(store.coeff.s>brek[i] &

store.coeff.s<=brek[i+1],

arr.ind=T)] <-valsi[i]

}

# if the data matrix is N x M, the color matrix must be (N-1) x (M-1)

colo2 <- colo1[-nrow(store.coeff.s), -ncol(store.coeff.s)]

lablist<-c("Ne=100000", "Ne=100","Ne=1000","Ne=10000","Ne=100000")

par(mar=c(4,4,1,1),bty="l",pty="s")

image(var.wanted,mean.wanted,Nepic,ylab=expression(italic("Mean age of onset")), col = gray((12:20)/20),

xlab=expression(italic("Variance in age of onset")))

contour(var.wanted,mean.wanted,Nepic,levels=pretty(Nepic),add=T,

labels=lablist,labcex=1.2)

box()

abline(h=max(mean.wanted)+0.15)

points(var.onset,mean.onset,pch=19, col="red")

legend(-25,70,legend="B",bty="n")
